# The mediating role of psychological capital in relations between spiritual well-being and mental health among nursing students

**DOI:** 10.1186/s40359-022-00935-0

**Published:** 2022-10-02

**Authors:** Ali Mohammad Parviniannasab, Mostafa Bijani, Ali Dehghani

**Affiliations:** 1 Department of Nursing, School of Nursing, Larestan University of Medical Sciences, Larestan, Iran; 2 Department of Medical Surgical Nursing, Fasa University of Medical Sciences, Fasa, Iran; 3 Department of Community Health Nursing, School of Nursing, Jahrom University of Medical Sciences, Jahrom, Iran

**Keywords:** Spiritual well-being, Psychological capital, Students, Nursing, Mediation effect, Mental health

## Abstract

**Background:**

Nursing students face mental and emotional issues due to the nature of their profession. The role of protective factors such as psychological capital and spiritual well-being is vital in improving mental health. This study investigated the mediating role of psychological capital as a mediator in the relationships between spiritual well-being and mental health in Iran.

**Methods:**

The present study was descriptive, cross-sectional research conducted on 426 undergraduate nursing students within a four-year educational program in Iran from July to December 2021. The participants were selected via convenience sampling. This research used psychological capital scale, spiritual well-being, and general health questionnaire. The collected data were then analyzed using descriptive tests, Pearson correlation, and a structural equation model.

**Results:**

Spiritual well-being positively affects mental health and psychological capital. Psychological capital also is positively related to mental health. Psychological capital partially mediated the effect of spiritual well-being on mental health.

**Conclusion:**

According to the results, High level of spiritual well-being can improve nursing students' mental health and the relationship is partially mediated Psychological capital. Therefore, psychological capital is an important factor in improving nursing students’ mental health.

## Introduction

Nursing as a profession faces many issues. Nursing students are more prone to mental and emotional disorders due to stressors such as the clinical and educational environment, death of patients, and psychological pressures of hospital [1, 2]. The findings show the increasing spread of mental disorders in this critical group, both in Iran and outside of Iran [3–5]. Therefore, health should be considered a multidimensional concept beyond the physical dimension. One of these essential and influential dimensions is spiritual well-being. Spiritual well-being can be defined as a sense of connection with others, meaning and purpose in life, faith, and a relationship with a transcendent power [6]. Ample research supports the hypothesis that spiritual well-being can enhance mental functioning [7–9].

It is interesting to note that the concept of psychological capital (PsyCap) has recently entered the academic field and can help improve the well-being of students. PsyCap consists of four components: self-efficacy, resilience, optimism, and hope [10]. Most PsyCap studies have focused on organizational employees [10], and few have examined students' PsyCap in academic settings. PsyCap has significant positive effects on job performance [10, 11], spirituality [4], mental health [12] and well-being [13]. Additionally, PsyCap was reported as a mediator between Negative life events and school adjustment [14], Job Stress, and Mental Health [15]. What is inferred from the above studies is that PsyCap can play a central role in the growth of an individual's well-being in all dimensions. The theoretical model for PsyCap inspires by positive psychology [16]. In this model**,** PsyCap, as a protective factor, was a positive resource for enhancing employees' well-being [13].

**Fig. 1:**
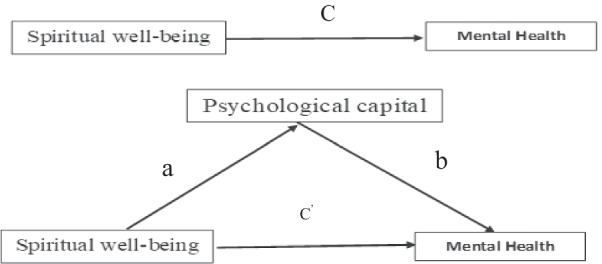
Hypothesized mediated model

Based on previous studies and the conceptual model mentioned, we propose that PsyCap may act as a mediating variable in the relationship between spiritual well-being and mental health among nursing students. Moreover, to our knowledge, the potential impact of spiritual well-being on PsyCap, and whether PsyCap mediates the relationship between spiritual well-being and mental health have not been examined in the context of Iranian nursing students Thus, we hypothesized that (1) spiritual well-being is positively correlated with Mental Health, (2) psychological capital is positively related to Mental Health, (3) Spiritual well-being is positively correlated with psychological capital, and (4) psychological capital mediates the relationship between Spiritual well-being and Mental Health (Fig. 1).

## Methods

### Study design and setting

A cross-sectional, descriptive design was conducted from July to December 2021. The specific aims were as follows: (1) to explore the associations between spiritual well-being, mental health, and PsyCap; and (2) whether PsyCap mediates the relationship between spiritual well-being and mental health. The study was conducted among undergraduate nursing students of four of the University of Medical Sciences in Fars province in the south of Iran. Students were invited to take part in the study. The inclusion criteria of the study were undergraduate nursing students who: were (1) willing to participate, (2) Completion of at least one academic semester, and (3) completion of the questionnaire. In the case of incomplete questionnaires, the samples were excluded from the study.

### Sample size and participants

Based on the study of Wolf et al. [17], to determine the sample size in structural equation modeling, he introduced a ratio of 5–10 samples per variable (number of questions). According to the number of 56 questions, a sample size between 280 and 560 seems desirable. 572 students participated in the sampling. Participants whose time to complete the questionnaire was less than 15 min were excluded from the study. Finally, 426 students participated in the study.

### Data collection

This study was approved by the Institutional Research Ethics Committee of Larestan University of Medical Sciences, Larestan, Iran (IR.LARUMS.REC.1400.018). Due to the COVID-19 pandemic, all the universities in Iran closed their facilities and transitioned to online teaching during data collection. Therefore, we carried out this study using an online questionnaire survey link. Participants were recruited through convenience sampling, using online-based survey links via social media platforms such as WhatsApp and student board pages. All participants were informed of the study aim and methods and assured of their anonymity and data confidentiality. Reminders were posted every four weeks to recruit as many students as possible. To prevent repetitive survey responses, students were not allowed to answer the questionnaire more than once from the same internet protocol address. The time to complete the survey is approximately 15 min. All data were stored on the researcher's computer with a password for the purpose of analysis.

### Instruments

#### Spiritual well-being (SWB)

The spiritual well-being scale was developed by Palutzian and Ellison [6]. The scale consists of two subscales of religious well-being (**RWB**) and existential well-being (EWB). Ten items (odd number) measure RWB, and ten items (even number) measure EWB. Eight items (1, 2, 5, 6, 9, 12, 13, 16) are adverse questionnaires. The scales are rated from 1 (strongly disagree) to 6 (strongly agree). The total score ranges from 20 to 120, and the two subscale scores range from 10 to 60. Cronbach's α in the original version were 0.91, 0.91, and 0.93 for RWB, EWB, and full scale, respectively [6]. Internal consistency of the Persian version was 0.82, 0.87, and 0.90, respectively [18]. In this study, Cronbach's alpha coefficients were 0.88, 0.89, and 0.93 for RWB, EWB, and the total scale, respectively.

#### Psychological capital scale (PsyCap)

The psychological capital scale (PsyCap) developed by Luthans [10] utilized in this study, consists of four subscales, namely self-efficacy (6 items), hope (6 items), resilience (6 items), and optimism (6 items). The scales are rated from 1 (strongly disagree) to 6 (strongly agree). The total score ranges from 24 to 124. Cronbach’s α in the original version were 0.72, 0.71, 0.75, 0.74, and 0.88 for hope, resilience, self-efficacy, optimism, and total scale, respectively [10]. Cronbach’s α in the Persian version were 0.83, 0.73, 0.87, 0.70, and 0.89 for hope, resilience, self-efficacy, optimism, and full scale, respectively [19]. In this study, Cronbach's alpha coefficients were found as 0.79, 0.71, 0.86, 0.72, and 0.89 for hope, resilience, self-efficacy, optimism, and full scale.

### General health questionnaire

The General Health Questionnaire (GHQ) was developed by Goldberg [20]. In this study, GHQ-12 was used. The scale asks whether the respondent has recently experienced a particular symptom or behavior. The most common scoring method is the Likert scoring style (0–3). Each item is rated on a four-point scale (less than usual, no more than usual, rather more than usual, or much more than usual), giving a total score of 36. The internal consistency of the questionnaire was measured using Cronbach's alpha coefficient to test the reliability. The alpha of the whole sample was 0.87 [20]. The validity of the Persian version for the entire GHQ was 0.96 [21]. In this study, Cronbach's alpha was 0.85.

### Data analysis

Descriptive statistics and correlation analysis of spiritual well-being, psychological capital, and mental health were analysed using IBM SPSS v24.0. A structural equation model (SEM) with AMOS 24.0 maximum likelihood estimation was used to test the hypothesized model. To examine the model fit, multiple fit indices, including the relative chi-square statistic (χ^2^/DF), goodness of Fit Index (GFI), adjusted goodness-of-fit index (AGFI), comparative fit index (CFI), normed fit index (NFI), Tucker–Lewis index (TLI), and the root mean square error of approximation (RMSEA) were used. In the present study, the following criteria were used to testify whether the model was fit: χ^2^/*df* < 3.00, NFI, GFI, AGFI, and CFI should be greater than 0.90, and RMSEA should be less than 0.05 [22]. To determine the mediating role of psychological capital, we used the bias-corrected bootstrap 95% confidence interval (CI) based on 5000 bootstrapping if the confidence intervals did not contain zero value, demonstrating statistical significance. The significance level was set at 5% (*P* < 0.05).

## Results

### Participants' characteristics

Table 1 shows the socio-demographic information of nursing students. Most participants were female (n = 236, 55.4%) and the mean age was 21.39 years (SD = 2.04). The students were in different academic years (year 1: 27.7%, year 2: 28.4%, year 3: 25.6%, and year 4: 18.3%). Regarding marital status, 393 participants (92.3%) were single (Table 1).

**Table 1:** Social-demographic information of nursing students (*n* = 426)

Variables	N (%)	%
Age (SD)	21.39 (Mean)	2.04
Gender		
Female	236	55.4
Male	190	44.6
Academic year		
Year 1	118	27.7
Year 2	121	28.4
Year 3	109	25.6
Year 4	78	18.3
Marital		
Single	393	92.3
Married	33	7.7

### Correlations between spiritual well-being, psychological capital, and mental health

Table 2 shows the correlations between studied variables. Spiritual well-being had a positive impact on mental health, with a significant correlation (*r* = 0.456, *p* < 0.01). A positive correlation was found between psychological capital and mental health (*r* = 0.464, *p* < 0.01). There was another significant positive correlation between spiritual well-being and psychological capital (*r* = 0.623, *p* < 0.01).

**Table 2:** Correlations, means, and standards deviations of study variables

Variable	1	2	3	M	SD	Skewness	Kurtosis
1. Mental health	–			19.87	5.55	− 0.579	− 0.140
2. Psychological capital	0.464**	–		83.84	14.08	− 0.611	0.965
3. Spiritual well-being	0.456**	0.623**	–	74.05	15	− 0.056	− 0.427

**p < 0.01 (2-tails)

### Path analysis of effects of spiritual well-being on mental health

**Fig. 2:**
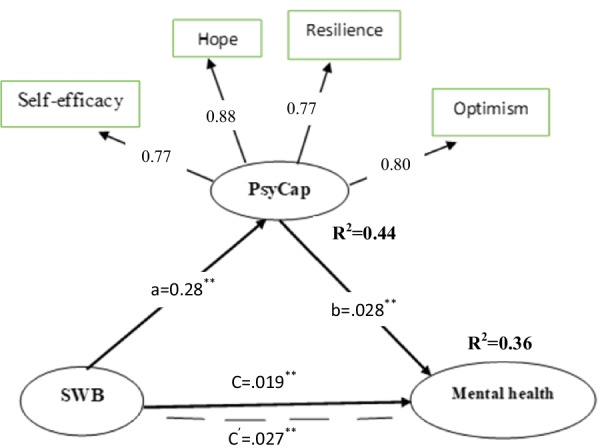
Final model. **c**: The total effect of Spiritual well-being on Mental Health without considering any mediator; **c**′: direct effect of Spiritual well-being on the Mental Health while considering role of the psychological capital; ***a***: effects of the Spiritual well-being on the psychological capital; ***b***: effects of the psychological capital on the Mental Health. **p < 0.01 (2-tails)

We conducted a confirmatory factor analysis (CFA) in the first step to test the structural model. The fit level of the modelling was acceptable with χ^2^/*df* = 2.12, GFI = 0.94, AGFI = 0.91, CFI = 0.96, NFI = 0.93, TLI = 0.95 and, RMSEA = 0.052 (Table 3). SEM verified the mediation hypothesis based on the overall mediating model in the second step. The results (Fig. 2; Table 4) show that spiritual well-being positively and significantly predicts mental health (*B* = 0.019, t = 5.54, *p* < 0.01), which supports Hypothesis 1. Psychological capital positively and significantly predicts mental health (*β* = 0.028, t = 3.55, *p* < 0.01), which supported Hypothesis 2. Spiritual well-being positively and significantly predicts psychological capital (*β* = 0.28, t = 10.79, *p* < 0.01), which supported Hypothesis 3.

**Table 3:** Index evaluation system and fitting results of overall structural equation model

Model fit	χ^2^	*df*	χ^2^/*df*	GFI	AGFI	TLI	CFI	NFI	RMSEA
Value	208.55	98	2.12	0.94	0.91	0.95	0.96	0.93	0.052
Acceptable-fit	–	–	< 3	> 0.90	> 0.90	> 0.90	> 0.90	> 0.90	< 0.08

**Table 4:** Total, direct, and indirect effects of each path in this model

Effect	Pathway	Estimated	BC 95% CI	
			Lower	Upper
		*β*		
Total effect	c	0.019**	0.013	0.027
Indirect effect	ab	0.008**	0.004	0.012
Direct effect	Ć^′^	0.027**	0.021	0.034

**p < 0.01 (2-tails)

### Mediational analysis

We used the bias-corrected bootstrap 95% confidence interval based on 5,000 bootstrapping to test the mediating effect [23]. Spiritual well-being had an indirect effect (β = 0.008, *p* < 0.01) on mental health mediated by PsyCap, with 95% CI of 0.004–0.012. Since this CI did not contain the value of 0, it represents a significant difference. The direct effect of spiritual well-being on mental health was 0.027 (*p* < 0.01), so the mediating effect of psychological capital was partial. Therefore, since both the indirect path (Spiritual well-being—PsyCap—mental health) and the direct path (Spiritual well-being—mental health) became significant, we can conclude that only part of the impact of spirituality on mental health was accounted for by psychological capital. The total effect of spiritual well-being on mental health was 0.019. The whole model explains 36% of all variance in mental health. The values of the total, direct, and indirect effects in the model are shown in Table 4.

## Discussion

The present study investigated nursing students' spiritual well-being, mental health, and psychological capital. The results of the data analysis support the study hypotheses showing that higher spiritual well-being can improve mental health directly and indirectly through PsyCap.

Firstly, the results of this study confirm its first hypothesis, which states that there is a positive and significant correlation between spiritual well-being and mental health. In other words, students' higher levels of spiritual well-being correlate positively with better psychological health. These findings are consistent with the results of other studies showing that daily spiritual practices, religious support, and religious and spiritual self-evaluation in one's life can significantly predict mental health and well-being [8, 24, 25]. Spiritual well-being causes adaptation, integration of inner and spiritual life with external life and work environment, psychological well-being, and growth of human health [26]. A study by Unterrainer et al. [27] showed that spiritual well-being and religion have a significant relationship with various mental health and personal aspects. Spirituality expresses essential elements of human nature [27]. The results of other studies on students showed that promoting religious beliefs reduces anxiety and depression among nursing students and play a preventive role against mental disorders [28, 29]. Spirituality helps promote manta health by providing a framework for describing life experiences and creating a sense of oneness and existential connection. People with spiritual and religious beliefs can cope with stress and psychological problems [24].

Secondly, the results of this study confirm the second hypothesis, which states that there is a positive and significant correlation between psychological capital and mental health. The four components of PsyCap (hope, optimism, self-efficacy, resilience) allow people to experience less vulnerability, more positive emotions, and more opportunities for success [30]. People with high PsyCaps find an adaptive coping mechanism for fighting depression and anxiety. However, they face more tension [31]. PsyCap makes people more resilient, adaptable, optimistic about the future, and confident in their abilities [32]. University instructors need to focus on aspects of PsyCap in the university curriculum. Psychological capital can be a valuable resource for students, helping them stabilize their education psychologically and physically healthier [33]. Krasikova et al. [12] reported, when the level of PsyCap decreases, psychological disorders such as PTSD, anxiety, depression, and substance abuse (alcohol and drugs) are increased. Also, previous studies have increasingly shown a negative correlation between PsyCap and factors such as stress, anxiety, workplace frustration, and burnout [12, 34, 35]. Previous studies found a positive and significant relationship between the components of students' psychological capital and well-being, life satisfaction, and experience of positive emotions [36, 37]. Therefore, according to the results of the present study, it seems that PsyCap among students is much needed, and previous studies have confirmed that PsyCap constructs are fundamental in coping with academic stress. So, it is necessary to direct interventions on psychological capital for nursing students, particularly in terms of hope for the future, resilience, self-efficacy, and improved psychological health.

Thirdly, the results of this study confirm the third hypothesis, which states that there is a positive and significant correlation between spiritual well-being and psychological capital. Students with higher spiritual well-being reported higher levels of self-efficacy in both dimensions of spiritual well-being. These findings are consistent with the results of other studies [38, 39].

The results of a study carried out by Asgari [38] showed a significant positive relationship between hope and the spiritual well-being of students, which is consistent with the present study. It can be said that religion creates a coherent belief system that makes people find meaning in their lives and hope for the future [40, 41]. In a study by Ghahremani and Nadi [42], there was no significant relationship between hope to the end, spiritual excellence, religious support, and religious practices. This discrepancy can be in terms of cultural and religious differences and the type of rituals. It can be said that religious beliefs give people the confidence that there is always a strong force behind them. Relying on their ideas, these people deal with events more quickly and are more hopeful and optimistic about the future [43]. Other studies show that spiritual well-being, one of the dimensions of health, can make a fundamental change in human beings [4, 44]. Also, a Gnanapraksh [45] study reported that spiritual well-being is directly related to reducing perceived stress, increasing effective coping methods, and increasing student resilience levels.

In explaining the relationship, it can be said that when a person is psychologically in a state of optimism, self-efficacy, resilience, and hope, there could be a higher mental capacity to establish coherence and harmony in the meaning, purpose and higher worldly values of life. In other words, taking advantage of spiritual well-being while increasing the psychological empowerment of individuals will put them in a more favorable position in terms of psychological capital.

Fourthly, the results of this study confirm the fourth hypothesis, which states that psychological capital partially mediates the relationship between spiritual well-being and mental health.

We explained that psychological capital improves the effect of spiritual well-being on mental health. In other words, people with higher spiritual well-being are likely to have higher psychological money, which can enhance mental health. In several studies, psychological capital has been a mediating variable [14, 30]. Another study showed that PsyCap partially mediates the relationship between adverse life events and university stress coping [14]. The results showed that PsyCap is a positive source for dealing with daily stressful events and positively affects university adaptation among nursing students [14]. A high level of psychological capital can affect a person's coping method in response to changes and environmental conditions related to mental health, which helps maintain their mental health [46]. Spirituality strengthens psychological capital by creating a sense of connection to the world and a broad perspective on one's life and others [47]. Spirituality can also be said to be effective in acquiring and promoting the components of psychological capital by providing a framework for interpreting and describing life experiences and thereby providing a sense of existential cohesion and interconnectedness [48].

### Limitations

The present study has several limitations. First, using a convenience sample does not permit the generalization of these findings. Therefore, random sampling that guarantees a specific conception should be used. Second, the present study adopted a cross-sectional design. Thus, the causal relationships cannot be determined. Therefore, further longitudinal research is needed to test associations among the three variables to verify the causality among spiritual well-being, psychological capital, and mental health. The third limitation was the self-reporting method of data collection, which can affect the accuracy of the data collected by the participants. Forth, because all of the subjects in this study were Muslim, it is necessary to repeat this study in other cultures. Finally, other variables that may affect mental health, such as academic burnout, have not been considered. Further research will be needed to identify the effects on mental health using additional variables.

### Strengths

Despite the mentioned limitations, the study used a structural equation model. The other strengths are examining nursing students' relationships between spiritual well-being, psychological capital, and mental health among nursing students. This adds to the knowledge of how spiritual well-being and psychological prosperity can modulate mental health.

## Conclusion

The results of the present study indicate that, on the one hand, spiritual beliefs can increase psychological capital and its components, namely self-efficacy, resilience, hope, and optimism; on the other hand, it is possible to help promote the mental health of students by improving their psychological capital. Thus, university instructors are suggested to use interventions designed to promote the four components of the psychological worth of nursing students to enhance their mental health.

## Data Availability

The datasets generated and/or analysed during the current study are not publicly available due to the necessity to ensure participant confidentiality policies and laws of the country but are available from the corresponding author on reasonable request.

## Abbreviations

RWB: Religious well-being
EWB: Existential well-being
SWB: Spiritual well-being
PsyCap: Psychological Capital Scale
GHQ: General Health Questionnaire
SEM: Structural equation model
AMOS: Analysis of moment structures
χ^2^/DF: Chi-square divided by the degrees of freedom
GFI: Goodness of fit index
AGFI: Adjusted goodness-of-fit index
CFI: Comparative fit index
NFI: Normed fit index
TLI: Tucker–Lewis index
RMSEA: Root mean square error of approximation
CI: Confidence interval
